# Hospital Notes

**Published:** 1894-07

**Authors:** 


### Hospital Notes.

AT A recent meeting of the trustees of the Woman's Hospital and Foundlings' Home, Detroit, the following resolution was adopted :

WHEREAS, The arrangement providing that nurses from the Correspondence School of Health and Hygiene be allowed to practise in the Woman's Hospital and Foundlings' Home was made without due consideration ; therefore,

*Resolved,* That such arrangement be and is hereby discontinued from this date, and that a resolution to the effect that no relation whatever exists between the said Correspondence School of Health and Hygiene and the Woman's Hospital and Foundlings' Home be published in the medical journals already specified.

